# Supplementary material for: Virtual reality for 3D histology: multi-scale visualization of organs with interactive feature exploration
Source: BMC Cancer. 2021 Oct 22;21:1133. doi: 10.1186/s12885-021-08542-9 (PMC8539837; doi:10.1186/s12885-021-08542-9)
Supplement: Supplementary file 1 — Additional file 1: Supplementary Video 1. Properties of the virtual reality application, including navigation in both organ and tumor level, and various functionalities and features are demonstrated in Supplementary Video S1. Supplement available at: https://github.com/BioimageInformaticsTampere/The-Virtual-Prostate. [file 12885_2021_8542_MOESM1_ESM.docx]

<https://github.com/BioimageInformaticsTampere/The-Virtual-Prostate>
